# A pilot study with early adolescents: dealing with diet, tobacco and air pollution using practical experiences and biological markers

**DOI:** 10.1186/s40248-017-0111-6

**Published:** 2017-12-01

**Authors:** Chiara Marabelli, Elena Munarini, Micaela Lina, Roberto Mazza, Roberto Boffi, Cinzia De Marco, Ario Ruprecht, Giorgia Angellotti, Chiara Veronese, Paolo Pozzi, Eleonora Bruno, Giuliana Gargano, Adalberto Cavalleri, Giulia Garrone, Franco Berrino

**Affiliations:** 1 Tobacco Control Unit - Fondazione IRCCS Istituto Nazionale Tumori, Via Venezian 1, 20133 Milan, Italy; 2 Pulmonary Rehabilitation Unit, Azienda Sociosanitaria Lariana, Sant’Antonio Abate Hospital, Via Domea 4, 22063 Cantù, Italy; 3 Department of Predictive & Preventive Medicine, Fondazione IRCCS Istituto Nazionale Tumori, Via Venezian 1, 20133 Milan, Italy; 4 Department of Biomedical Sciences for Health, University of Milan, Milan, Italy

**Keywords:** Prevention, Smoking and tobacco use, Tobacco control and policy, Dietary, Pollution, School-based, Biological markers, Obesity

## Abstract

**Background:**

Tobacco use and the Western diet are two of the most important and investigated topics in relation to adolescents’ health. In addition, air pollution is a crucial subject for future generations. School is a key social environment that should promote healthy behaviors in children and adolescents. In this field many different programs have been conducted, with mixed results and effectiveness. Research data suggest that comprehensive and multicomponent approaches may have a greater effect on tobacco use and diet, especially when integrated into a community-wide approach.

**Methods:**

The present work describes a multi-area pilot study called “La Scuola della Salute” (the School of Health) with a focus on the methodological aspects of the intervention. In our study we assessed different web-based and practical experiences related to adolescents’ smoking and dietary behaviors and awareness of smoke-related air pollution. Furthermore, to make adolescents more conscious of smoking and dietary behaviors, we conducted experiential workshops that addressed smoking and environmental pollution, food education, and lifestyle. Teachers and school administrators were involved in the project.

**Results:**

At baseline we investigated dietary habits, tobacco use, and individual and social characteristics by means of lifestyle questionnaires. In addition, we collected anthropometric parameters and performance indicators such as exhaled carbon monoxide and urinary fructose to assess smoking and nutrition habits. At the end of the intervention lifestyle questionnaire and biological markers were collected again: knowledge about these topics was significantly improved, and the urinary fructose was able to estimate the levels of obesity in the classes.

**Conclusions:**

The integrated approach, combined with the use of biological markers, could be an innovative approach to the promotion of healthy lifestyles among adolescents, but further research is needed.

## Background

Tobacco use and the Western diet are two of the most important and most extensively investigated topics related to the health of adolescents and the prevention of any consequent illness during their lifetime. Furthermore, air pollution is a crucial theme for future generations. Tobacco use is currently the leading preventable cause of death worldwide [1], and reduction of the smoking prevalence among adolescents is a public health priority for several reasons [2]. Adolescents risk developing lifelong smoking behaviors [3]. The 1994 Surgeon General Report on preventing tobacco use in young people pointed out that cigarette smoking is the strongest addictive behavior, tending to strengthen during adolescence, and that people who begin to smoke at an early age develop a nicotine addiction more than those who start at a later age [2, 4]. At the same time, overweight and obesity in children and adolescents are becoming major global public health concerns [5–8].

The major cause of obesity and overweight is an energy imbalance between consumed and spent calories. In particular, increased consumption of sugar-sweetened beverages and sweets ([9–12]; chips [12], energy-dense and processed foods [12, 13] on one hand and decreased physical activity [14, 15] on the other hand induce long-term weight gain in children/adolescents and adults. Furthermore, in children and adolescents, the amount of time spent in front of the television or computer or playing videogames may influence energy consumption, energy expenditure, or both [16, 17]. Obesity during childhood and adolescence is associated with a higher probability of obesity during adulthood [18], premature death, disability [19, 20] and psychological problems.

Additionally, research into the individual and combined factors that influence our choices related to health, such as tobacco use and type of diet, indicates that various unhealthy habits may in some way be associated, and that these habits start with adolescence. Even if it is unclear how the Western diet, tobacco use and secondhand smoking together impact on the risk of chronic disease, their synergistic action on health may significantly increase this threat to a greater level than any of these factors alone [21, 22].

School seems to be a fundamental setting to educate children about smoking behavior risks and implement anti-tobacco policies, but also to provide and promote nutritional education. The perception of smoking restrictions in the school is correlated with lower rates of tobacco use among adolescents [23–26], as well as their perceived general connectedness with school context [27].

The International Agency of Research on Cancer of the World Health Organization (WHO) classified in 2013 outdoor air pollution as carcinogenic to humans; the most recent data indicate that in 2010, 223,000 deaths from lung cancer worldwide resulted from air pollution [28].

Although the WHO definition of health as “complete physical, mental and social well-being” (WHO, 1946) goes back more than 50 years, few studies [21, 22] have combined different areas of intervention in school-based prevention programs and their effects on adolescents’ lifestyles, and none ever included environmental education.

In this paper we will first review the literature on these topics and then present our multi-area experiential school-based intervention “La Scuola della Salute”, aimed at exploring a new way to talk with adolescents about smoking, nutrition and pollution. Care for the environment can be a useful perspective to start talking with adolescents about their health; that is why we decided to include air pollution in the intervention, in addition to tobacco use and the Western diet.

## Methods

This intervention protocol was approved by the ethics committee of Istituto Nazionale dei Tumori (INT), Milan. From February to September 2011 the project was defined and the schools were selected and prepared for the intervention through meetings conducted by the project team.

The intervention began at the end of September 2011 and ended in March 2012. Before attending any of the experiential workshops, students filled in anonymous questionnaires regarding personal data, psychological characteristics, and their habits and knowledge concerning smoking and diet. After the questionnaires had been completed, all teachers and students received lifestyle guides. At the end of the study students filled in follow-up questionnaires regarding diet and smoking, and their knowledge about these subjects was tested. Every student was assigned a personal alphanumeric code (composed of subject number, sex, grade class and order of measurement) so that his or her answers could be linked to the final questionnaire and to all data collected in the study. At the end of the school year, students were informed about the general results of the questionnaires.

### Food education course

The primary aim of the food education course was to offer practical and cognitive tools to help students develop a more responsible and conscious relation to food and to a healthy diet and lifestyle. The secondary aim was to devise an analytical method that allowed to estimate any change in dietary sugar intake before and after the intervention based on the measurement of urinary fructose. The food education course included a theoretical and a practical part. At the end of the course anthropometric measurements (weight and height) were made and urine samples were collected.

### Smoke and environmental pollution experimental laboratory

To make adolescents aware of the effect of direct and secondhand smoke and the pollution it causes, scientific instruments were used and brief experiments were carried out.

#### Measurement of carbon monoxide in exhaled air (ex-CO) and tobacco smoke

Measurement of ex-CO in breath was used to show what happens in smokers. The analyzer (see Instruments section) was operated by personnel expert in the procedure. We used this instrument to make adolescents aware of the impact of smoke on their bodies, and to biologically assess the degree of physical tobacco dependence. Students were invited to measure ex-CO themselves in order to practice with the analyzer and understand the impact of smoking. In addition, the CO level in the smoke plume of a cigarette was measured at a few centimeters from the cigarette. The analyzer display immediately signaled very high levels of CO.

#### Air quality assessment

Fine particulate matter, black carbon and organic carbon were measured during this workshop. The pollutants’ analyzers were connected to a wall screen initially showing indoor background concentrations (typically <50 mcg/m^3^), which rose enormously after a cigarette was lit in the room. These experiments were indicative of the vast quantity of particles emitted by a cigarette, and of the high level of pollution due to secondhand smoke.

#### Tobacco smoke workshop. Measurement of tobacco smoke pollution in a park

Real-time measurement of particulate pollution (PM1, PM2.5 and PM10) with portable instruments allows students to recognize that cigarettes can result in marked exposure to passive smoking even in outdoor environments such as parks. Workshops were held with groups of students representing each grade class.

### Behavioral and lifestyle workshops

The aim of these workshops was to prompt students to reflect upon and be more conscious of their behaviors related to health under the guidance of a psychologist. The behavioral and lifestyle experiential workshops also served as a connection between all the workshops of the project. Students’ opinions, observations and criticisms about the other workshops were discussed in order to learn if and how the activities proposed had an influence on the students’ lifestyles. Photo collage and role playing methods were used.

### Other

A website, Facebook page and YouTube channel were created to involve students and inform them about the project. Students were directly involved in two press conferences regarding the project. Students and teachers were given two different guides about smoking and diet.

## Results

### Sample description

Out of a total of 306 students, 277 (68% female, 90% aged 13–15 years) were enrolled in the study. The remaining 29 students did not return the informed consent form signed by their parents and could, therefore, not participate. Thirty-five percent of the adolescents attended humanities-oriented secondary schools, 24.9% attended science-oriented secondary schools, and 40.1% attended didactics and education-oriented secondary schools. Table 1 summarizes the sample divided by sex, age and school type.

**Table 1:** Sample description

	No.	Percent
Sex		
Female	188	67.9
Male	89	32.1
Age (years)		
≤ 15	250	90.3
≥ 16	27	9.7
School type		
Humanities	97	35.0
Sciences	69	24.9
Didactics and education	111	40.1

#### Participants’ smoking habits

Table 2 reports the total sample data related to smoking, Table 3 reports the data of current smokers, and Table 4 presents the ex-CO data. We reported some missing values in the collection of the data because the students did not answers all the questions related to smoking, even when they declared themselves as smokers. At the same time, subjects who didn’t consider themselves as current smokers answered the question related to the first cigarette as well because they had tried to smoke in the past.

**Table 2:** Total sample data related to smoking

	No.	Percent
Have you ever smoked?		
Yes	86	31.9
No	184	66.4
Age at first cigarette (years)		
**≤** 13	36	42.2
**≥** 14	49	57.6
First cigarette smoked with whom?		
Friend	77	91.7
Alone	3	3.6
Family member	4	4.8
Cigarettes per day		
**≤** 6	36	66.7
**≥** 7	18	43.3
Why did you start?		
Curiosity	44	67.7
My friends were smoking	5	7.7
Other	16	24.5
With whom do you smoke?		
Alone	2	3.6
With friends	23	41.8
Indifferent	30	54.5
Do you think it’s easy to stop?		
Yes	25	42.4
No	34	57.6

**Table 3:** Current smokers’ data

	No.	Percent
Sex		
Male	12	24
Female	38	76
Cigarettes per day		
≤ 6	32	64
≥ 7	18	30
Why do you keep smoking?		
I like it	23	46
Unable to stop	7	14
Better concentration	2	4
I feel more self-confident	3	6
My friends smoke	3	6
Other	12	24

**Table 4:** Exhaled CO data

	No.	Ex-CO* at T1	P
Smoking status			
Not smoking	209	0.43 (± 0.83)	t(45.408) = 5.61, *p* < 0.01
Current smokers	45	3 (± 3.05)	
Sex			
Male	82	0.56 (± 1.03)	t(250.85) = −2.47, *p* < 0.05
Female	172	1.03 (± 2.02)	
Sex/Current smokers			
Male	9	2.33 (± 1.58)	t(43) = −0.72, *p* = 0.47
Female	36	3.17 (± 3.3)	

*Ex-CO data are expressed in parts per million (ppm)

Thirty-one percent (*n* = 86) of the total sample had smoked at least once. Out of the students who answered these questions, 42.2% had their first cigarette before age 13, 91.7% smoked for the first time in the company of a friend, and 68% tried smoking out of curiosity. Out of the smokers among the students, 66.7% smoked fewer than six cigarettes per day (Table 2).

Baseline reported smoking was more frequent among girls (20%) than boys (13%), with a mean ex-CO test of 1.03 ppm in girls and 0.56 in boys (*p* < 0.05) (Table 4). Total sample ex-CO at baseline was 0.86 ppm and the maximum value registered was 13 ppm (in one student only).

We considered as current smokers those students who declared being smokers in the questionnaires, amounting to a total of 50 students. Among these, 45 were tested with the Ex-CO, while five of them missed the test. We observed a significant difference (*p* < 0.01) in ex-CO between current smokers (mean 3 ppm; *n* = 45) and the rest of the sample (mean0.43 ppm; *n* = 209) (Table 4). Among the current smokers (*n* = 50; females = 38, males = 12), 46% (*n* = 23) said they continued smoking because they liked it, and 16% (*n* = 7) because they were unable to stop. Thirty percent (*n* = 18) of current smokers smoked more than seven cigarettes per day (Table 3). It did not matter to them who they smoked with (54.5% considered this indifferent) and 61.2% (*n* = 30) would like to quit. Thirty-five current smokers tried to quit and 66% (*n* = 34) thought it was not easy. Ex-CO was higher in the girls group (m = 3.17 ppm, max = 13) than the boys group (m = 2.33 ppm; max = 5).

We observed a significant association (χ^2^(1) = 18.35, *p* < 0.01; *n* = 276) between being a current smoker and living with at least one family member who smoked. In the group of nonsmokers, 42% (*n* = 116) did not live with a smoking family member; in the group of current smokers, only 3.3% (*n* = 9) did not live with a smoking family member.

#### Participants’ dietary habits

We included 251 students with complete baseline data on lifestyle, anthropometric and dietary variables. The baseline characteristics of the students, divided by sex and grade class, are reported in Table 5. Forty-nine (19.5%) students were overweight (INRAN guidelines, 2003) [29]. The proportion of overweight adolescents was significantly higher among females than males: 10.4% of females and 9.1% of males had a BMI higher than 24 (mean 26.9 ± 3.6) and 22.5 (mean 25.0 ± 3.6), respectively (Table 6). Overweight increased with age and in the higher grades it was more frequent in males than females (11.0% vs 9.5%). Twelve urine pools were collected (one pool per class). Baseline values of urinary fructose were normally distributed and there were no significant differences between classes. The mean concentrations overall and divided by class are reported in Table 6.

**Table 5:** Baseline food and lifestyle characteristics of students by sex and grade class

	Male (*n* = 82)	Female (*n* = 169)	*P*	9^th^ grade (*n* = 124)	10^th^ grade (*n* = 127)	P
Sweetened beverages	8.6 ± 8.9	7.9 ± 9.4	0.17	8.9 ± 10.1	7.5 ± 8.4	0.23
Sweet snacks	10.4 ± 10.2	7.2 ± 7.1	<0.01	8.1 ± 8.8	8.4 ± 8.1	0.76
Salty snacks	4.8 ± 6.6	4.1 ± 5.1	0.32	3.9 ± 4.2	5.1 ± 6.4	0.08
Sport	2.8 ± 1.4	2.4 ± 1.2	0.05	2.5 ± 1.1	2.6 ± 1.5	0.77
TV (hours/day)	2.9 ± 1.1	2.6 ± 1.1	0.02	2.6 ± 1.1	2.8 ± 1.1	0.38

**Table 6:** Baseline anthropometric characteristics of students by sex and grade class

	Sex	Class		
	Male (n = 82)	Female (n = 169)	9^th^ grade (n = 124)	10^th^ grade (n = 127)
Weight (kg)	62.6 ± 11.0	54.9 ± 10.1	56.0 ± 11.5	58.8 ± 10.4
Height (cm)	171.8 ± 7.6	161.2 ± 5.8	162.8 ± 7.6	166.6 ± 8.2
BMI	21.1 ± 3.1	21.1 ± 3.4	21.0 ± 3.5	21.1 ± 3.1
Overweight (%)	9.16	10.4	18.6	20.5
Mean urinary fructose of class pool (μg/mL)	2.4 ± 2.04		2.4 ± 2.8	2.4 ± 1.16

Table 7 describes the baseline food and lifestyle characteristics of the study population. On average, in 1 week, students consumed more than one can of sugar-sweetened beverage per day (8.2 ± 9.3 portions a week). The intake rate was higher in males than females and decreased with age. Furthermore, students consumed one or more sweet snack portions per day (8.2 ± 8.2 portions a week). The weekly intake rate was significantly higher in males than females (*p* < 0.01) and increased with age (*p* = 0.76). Intake of salty snacks was lower than that of the aforementioned energy-dense foods, with an average intake of four to five portions a week. Intake was higher in males (*p* = 0.35) and among 10^th^-grade students.

**Table 7:** Baseline food and lifestyle characteristics of students by BMI

	Normal weight (*n* = 202)	Overweight (*n* = 49)	P
Sweetened beverages	8.6 ± 9.8	6.3 ± 6.3	0.11
Sweet snacks	8.5 ± 8.6	7.3 ± 7.4	0.36
Salty snacks	4.1 ± 5.0	5.3 ± 7.8	0.18
Sport	2.5 ± 1.2	2.8 ± 1.7	0.14
TV (hours/day)	2.7 ± 1.1	2.7 ± 1.2	0.89

*P* in Student’s t-test

With regard to physical activity and sedentariness, 76% of students, in addition to physical education at school, practised physical activity on average 2.7 times a week. Males were significantly more active than females (*p* = 0.02), but they also spent significantly more time watching television or playing videogames (*p* = 0.04).

Table 7 describes the baseline food and lifestyle characteristics of students by BMI (normal weight vs overweight). We observed that overweight students declared they consumed fewer portions of sweetened beverages and sweet snacks, and claimed to be more active than normal-weight students. They consumed more salty snacks and there were no differences in the time spent in front of the television or computer.

The prevalence of overweight and urinary fructose levels was measured for each class. We could not measure urinary fructose in every student, but when we stratified students by the median overweight prevalence (≤14.3% vs >4.3%), we observed higher levels of fructose in the classes with a higher prevalence of overweight (1.76 ± 1.23 versus 2.87 ± 2.46 μg/mL, *p* = 0.56).

#### Participants’ smoking and dietary knowledge

As regards their initial knowledge about smoking and dietary habits, students reached on average a score of 3.77 (± 1.34) and 3.09 (± 1.36), respectively. The maximum score obtainable was 10 on either questionnaire.

### Efficacy indicators

#### Measurement of CO in exhaled air

CO measurement was done with a double aim. The primary aim was to give young smokers a practical experience of the consequences of smoking: they could see with their own eyes the impact of smoking in terms of CO increase and oxygen decrease in the body. The secondary aim was to assess the adolescents’ smoking status.

#### Measurement of dietary added sugar (urinary fructose)

We decided to take the urinary fructose level as a performance indicator. The necessity of a biological biomarker arose because dietary interviews or questionnaires are known to be prone to measurement errors [30].

The following results were registered 3 months after the beginning of the intervention.

### Knowledge about smoke, pollution and nutrition

The mean score of knowledge about smoke and pollution increased from 3.77 (±(SD 1,34) to 4,.53 (SD ±1,55) after the intervention (*N* = 265), with a significant paired samples t-test (*p* < .001). Knowledge score about nutrition increased from 3.09 (SD ±1,36) to 3,.92 (±SD1,52) as well, with a significant paired samples t-test (p < .001) (Table 8).

**Table 8:** Differences in knowledge at T1 and T2

Knowledge	T1 (265)	T2 (265)	P
Smoke and pollution	3.77 SD 1.34	4.53 SD 1.55	<.001
Nutrition	3.09 SD 1.36	3.92 SD 1.52	<.001

### Ex-CO levels and smoking habits

Although exhaled CO measurement was carried out only with educational purpose, we registered decreasing trends from T1 (0.86 ppm, ±SD 1.73) to T2 (0.68 ppm, ±SD 1.91), n.s. (*N* = 258). Considering only the current smokers group (*N* = 43), the ex-CO level decreased from 2.98 (±SD 3.02) to 2.42 ppm (±SD 3.65), n.s.

The percentage of students who thought at T1 that quitting smoking is easy, decreased from 34 to 28.9% in the current smokers group, and from 42 to 35.6% in the whole sample (Table 9).

**Table 9:** Differences in exhaled CO at T1 and T2 in the total sample and in the current smokers group

Exhaled CO	T1	T2
Total sample (N = 258)	0.86 (SD 1.73)	0.68 (SD 1.91)
Current smokers (N = 43)	3.09 (SD 1.36)	3.92 (SD 1.52)

## Nutrition

Students reduced salty snack consumption rates (*p* = 0.03). Stronger differences were observed in 2^nd^ year students (−1.8, ±SD 5.9 vs 0.2, ±SD6.6; *p* = 0.01). We registered a significant reduction of TV hours per day (*p* < 0.05) as well. In the classes were a BMI reduction was observed, fructose level was significantly lower if compared to classes where BMI increased (p < 0.05 = 0.03; *R* = 0.68; p = 0.01). More relevant and significant changes were observed in the female group if compared to the male (Table 10).

**Table 10:** Differences in antropometrics and nutrition at T1 and T2 in the total sample

Consumption per week	T1 (251)	T2 (226)	P
Weight	57.2 (SD 10.4)	57.9 (SD 10.5)	<.001
Height	165.0 (SD 8.2)	166 (SD 0.54)	<.001
BMI	20.9 (SD 3.0)	21.0 (SD 3.0)	<.01
Sugar beverages	8.0 (SD 8.7)	7.2 (SD 7.6)	<.05
Sweet snacks	8.5 (SD 8.3)	7.8 (SD 6.6)	<.05
Saulty snacks	4.5 (SD 5.6)	3.6 (SD 4.8)	<.01
Sport	2.0 (SD1.5)	2.1 (SD 1.5)	N.S.
TV hours/day	2.7 (SD 1.1)	2.3 (SD 1.1)	<.001
% Overweight between boys and girls	19.5%	18.2%	<.001

### Urinary pools

Urines were collected with 141 subjects, grouped into 12 pools, one for each class. Subcjets affected by diabetes (*n* = 2) and insulin resistant (*n* = 1) were excluded.

For each class we studied the relation between being overweight and food consumption frequency. Linear regressions revealed a significant statistical relation between overweight and sweet and salty snacks consumption also if controlling the age (Table 11).

**Table 11:** Relation between overweight and nutrition

Adjusted for age	Coeff.	P	IC
Sweets	2.41	<.05	1.00–3.82
Sweetened beverage + sweet snacks	1.05	<.05	0.10–2.01
Chips	3.75	0.01	0.10–6.38

No significant relation was observed comparing fructose concentration before and after the intervention. In the classes where a reduction of BMI was observed, fructose levels significantly reduced as well, with an opposite tendency if compared to the classes were we registered an increased BMI and which showed a higher level of fructose (p = 0.03; R = 0.68; p = 0.01) Table 12.

**Table 12:** Fructose levels and IMC variations at T2

	IMC Reduction	IMC Increase	P*
Δ Fructose	−1.02 (SD 2.50)	2.18 (SD 1.86)	<.05

*P in Student’s t-test

## Discussion

The aim of this article is to describe and discuss a new method of intervention among secondary school adolescents that is aimed at promoting a healthy lifestyle. This pilot study is characterized by the application of multimodal integrated strategies: we integrated (a) prevention areas (smoking, diet and environment), (b) methods of intervention (experiential workshops, school courses, informative material, dedicated web platform, scientific research, media communications), and (c) different roles (students, teachers, parents, researchers, journalists, citizens).

- Consistent with a concept of health that includes the influence of multiple factors, recent research has focused on the integration of multiple health areas in prevention interventions. Furthermore, smoking and dietary prevention projects have already been conducted, but none of these included environmental pollution as part of the educational intervention. Many adolescents are informed about the health risks associated with smoking but rarely receive information about the content of cigarette smoke and how it influences the indoor and outdoor environment. The relation between smoking and pollution therefore brings new and more articulated considerations onto the prevention path.
- The aim of our multimodal approach is to stimulate adolescents to think about smoking and dietary concerns at different moments in their everyday life. The website, the public park research and the press conferences have the common goal of making adolescents actual partners in the project and social promoters of healthy changes.

Web instruments have the capacity to rapidly and easily connect people who are interested in these matters, and also bring us closer to the social market. Although there are studies in the literature that used web instruments in prevention interventions [31–33], their use in this field is recent, and this is another innovative and relevant aspect of the project described here.

(c) Finally, our project involved not only school components and families, but also the wider urban community. Adolescents perceptions and beliefs about tobacco use are the most proximal influence on their behaviour: for example, the number of family members and friends who smoke is strongly associated with youth smoking. The results of this paper confirm this finding. At the same time, to make students more involved in the wider living context, we wanted them to be interviewed and invited to take part in television broadcasts.

The choice to use biological markers to assess the samples’ characteristics and the impact of our intervention is an innovative aspect of this project.

Regarding the smoking area, it’s already assessed that Ex-Co is a reliable indicator of smoking status in adult and in several subgroups of smokers [34–36]. Despite this, few studies investigate if CO measurement among adolescents is an instrument able to discriminate smokers and non-smokers. In the Vançelik’s et al. study [37] was found that CO measurements between adolescents with a median age of 17 have a good sensitivity (90%) in assess the smoking status. Our results confirm Vançelik’s data: comparing the current smokers group to the rest of the sample, we observed a significant difference (*p* < 0.01) of the ex-CO values between the two groups. Further studies are needed to confirm these results in this smokers’ sub-group.

Regarding the dietary area, one of the methodological purpose of this pilot study is to test the feasibility to create a class pool of urinary fructose as biomarker for the estimation of dietary sugar intake. We decided to use a biological biomarker because typically interviews or dietary questionnaires are known to be prone to measurement errors [30].

So, to test the urinary fructose levels we decided to create a pool of urinary for each class in the study. All classes pools presented normal value of urinary fructose and we didn’t observe any significant differences between class grades. However, when we stratified each class by the prevalence of overweight we observed a higher level of fructose in the classes with a higher prevalence of overweight .

In the dietary area, urinary fructose was used to estimate dietary sugar intake because recent research [38] demonstrated that sugar consumption is a significant determinant of obesity, with a clear dose effect. So, to test the urinary fructose levels we decided to create a pool of urinary samples for each class in the study. The use of a single urinary sample pool as we did in this study allows to extrapolate the average situation of the individuals of that particular class. Therefore, a very limited number of analyses is needed to test dietary changes. This means saving considerable time and money for the execution of a analytical assays, especially in large epidemiological studies.

Considering the changes in the behaviors, Ex-CO and fructose levels showed interesting trends, but did not yield statistically significant results at the 3 months follow up. These results can be explained with the short exposure the students had to the intervention. Furthermore, 3 months is a too short time to answer the question whether the intervention is effective or not, or whether these biological markers are effective or not in the assessment with adolescents.

The study has several others limitations, first of all its challenging replicability. This project, in fact, needs spaces expressly equipped with instruments for particulate matter, black carbon and CO measurements and experiments, as well as large kitchens where students can cook healthy food. Furthermore, it requires (at least for the first edition) considerable financial resources for the purchase of instruments and equipment.

Secondly, we proposed our intervention within a limited time frame, while the literature suggests that repeated recalls and family involvement result in a more effective intervention. We could not carry out extended follow-up, and to fill the gap we integrated the themes of the project (pollution, diet, smoking) into the school curriculum so that teachers could refresh them during the following years, and stimulate students to follow the website.

Finally, the management of biological markers can be difficult in this age group, in particular urine collection, which involves a very intimate sphere during adolescence.

## Conclusions

Knonwledge about the topics was significantly improved and biological markers showed interesting results. The fructose levels collected also allowed us to estimate the levels of obesity in the classes. The integrated method of intervention we tested with adolescents needs to be replicated and further explored. However, the causes for reflection we received from this first application are very promising and suggest that this approach could have a greater impact in promoting healthy lifestyles among adolescents.
